# The Role of the Employer in Supporting Work Participation of Workers with Disabilities: A Systematic Literature Review Using an Interdisciplinary Approach

**DOI:** 10.1007/s10926-021-09978-3

**Published:** 2021-05-12

**Authors:** J. Jansen, R. van Ooijen, P. W. C. Koning, C. R. L. Boot, S. Brouwer

**Affiliations:** 1 Department of Health Sciences, Community and Occupational Medicine, University of Groningen, University Medical Center Groningen, Groningen, The Netherlands; 2 Department of Economics, Econometrics and Finance, University of Groningen, Groningen, The Netherlands; 3 Department of Economics, VU University Amsterdam, Amsterdam, The Netherlands; 4 Department of Economics, Leiden University, Leiden, The Netherlands; 5 Department of Public and Occupational Health, Amsterdam UMC, VU University Amsterdam, Amsterdam Public Health Research Institute, Amsterdam, The Netherlands

**Keywords:** People with disabilities, Return to work, Employment, Social support, Workplace

## Abstract

*Purpose* There is growing awareness that the employer plays an important role in preventing early labor market exit of workers with poor health. This systematic review aims to explore the employer characteristics associated with work participation of workers with disabilities. An interdisciplinary approach was used to capture relevant characteristics at all organizational levels. *Methods* To identify relevant longitudinal observational studies, a systematic literature search was conducted in PubMed, Web of Science, PsycINFO and EconLit. Three key concepts were central to the search: (a) employer characteristics, (b) work participation, including continued employment, return to work and long-term work disability, and (c) chronic diseases. *Results* The search strategy resulted in 4456 articles. In total 50 articles met the inclusion criteria. We found 14 determinants clustered in four domains: work accommodations, social support, organizational culture and company characteristics. On supervisor level, strong evidence was found for an association between work accommodations and continued employment and return to work. Moderate evidence was found for an association between social support and return to work. On higher organizational level, weak evidence was found for an association between organizational culture and return to work. Inconsistent evidence was found for an association between company characteristics and the three work outcomes. *Conclusions* Our review indicates the importance of different employer efforts for work participation of workers with disabilities. Workplace programs aimed at facilitating work accommodations and supervisor support can contribute to the prevention of early labor market exit of workers with poor health. Further research is needed on the influence of organizational culture and company characteristics on work participation.

**Supplementary Information:**

The online version contains supplementary material available at 10.1007/s10926-021-09978-3.

## Introduction

Several OECD countries reformed their disability programs over the past decades to foster labor market integration of people who face challenges staying or re-entering the workforce due to illness or disabilities [1]. These reforms primarily focused on the reintegration of workers with disabilities into employment; recognizing that many of them only have partially reduced work capacity and could therefore continue working if adequately supported by their employer [1–3]. Following these reforms the employment rates of people with disabilities has increased over the years [1, 4].This suggests that employment outcomes of people with disabilities are not only affected by their health conditions but also by their work environment [5].

As a result, there is growing awareness that the employers’ organizational context plays an important role in preventing early labor market exit of workers with poor health. The organizational context is defined as the characteristics of a workplace, including the social, physical and organizational structure of a company [6]. As such, both the employers’ disability management policies and practices and the social interaction between employers and employees may influence job retention of employees with disabilities [7]. An employer can, for instance, support employees with disabilities by offering workplace accommodations with the aim to improve job functioning, facilitate faster return to work, and remove job related barriers [8].

In occupational health care, several studies have been published about employer-related determinants and intervention strategies that improve labor market participation of workers with disabling health conditions. These studies in particular focus on workers with musculoskeletal disorders [9–12], mental health conditions [10, 13] and/or cancer [14, 15]. Besides company characteristics, supervisor support is often reported as an important employer-related determinant of return to work, however findings are mixed [9, 13, 14]. Employer-related intervention strategies in particular focus on workplace accommodations used by employers to recruit, hire, retain, and promote persons with physical disabilities, i.e. physical/technological modifications, accommodations to enhance workplace flexibility and worker autonomy and strategies to promote workplace inclusion and integration [16]. Rigorous evaluations of the effectiveness of these accommodations is not well-documented in peer reviewed literature yet [10, 16]. Economic studies, on the other hand, often focus on the overall effectiveness of work accommodations regardless of the cause, across all types of health conditions, and frequently focus on the costs and benefits of different return-to-work programs, to learn what program works best. Another strength of the economics field is their use of largescale register data, adding knowledge to the field of occupational health. Each discipline and its corresponding research methods thus provides different insights about employer efforts and work participation of workers with disabilities, making them complementary to each other. As the topic of employer support for workers with disabilities is being investigated by different disciplines, an interdisciplinary approach is crucial to obtain a complete overview.

Moreover, to get a better insight into the role of employers in supporting workers with disabilities to continue their jobs it is important take into account the role of the employer at all organizational levels. Rather than only focusing on work accommodations, as was the focus of previous reviews [16], we strive to include a broader range of employer efforts by integrating the existing evidence from different disciplines. Such an interdisciplinary approach requires a comparison of different types of work disabilities and work participation outcomes, because different outcomes and types of work disabilities are considered relevant in different disciplines. In addition, in contrast to other reviews we include longitudinal quantitative studies which allows us to summarize the evidence of the associations between prognostic factors at the employer level, and long-term work outcomes. Therefore, we will focus on three long-term work participation outcomes: return to work, continued employment and long-term disability. To date, such an integration of the existing evidence on prognostic factors at employer level from different disciplines has not been conducted.

Thus, this systematic review aims to explore the employer characteristics associated with work participation of workers with disabilities through an interdisciplinary approach including an occupational health, psychology and economic perspective.

## Method

### Search Strategy

We conducted an interdisciplinary search using four databases: Pubmed, PsycINFO, Web of Science and EconLit (inception of databases until 17 April 2018). Pubmed was selected for its coverage of health and medicine-focused journals. PsycINFO was selected for its coverage of journals with a focus on psychology. Web of Science was selected for its coverage of occupational health journals. EconLit was selected for its coverage of economic journals. The key concepts used in the search strategy were developed by the research team with the support of a university librarian with an expertise on making systematic review searches. Three key concepts were central to the search: (a) employer characteristics; (b) work participation; and (c) chronic diseases. Synonyms were identified for each concept, including keywords and phrases as well as database-specific subject headings (e.g. MeSH headings) (online supplementary text S1). The search terms were adapted to each database to best utilize the search functionality and controlled vocabularies unique to each of them.

### Selection of Studies

Two independent reviewers (JJ, RvO) performed the selection of the studies in three screening phases. In the first phase, articles were excluded based on titles and abstracts. The systematic reviews application Rayyan was used for the initial screening of titles and abstracts [17]. All peer-reviewed journal articles were screened according to pre-defined criteria by the research team: (i) the study population consisted of workers with a chronic disease; (ii) the subjects were aged 18–67 years (i.e., working age population); (iii) the study used a longitudinal quantitative study design; (iv) the study examined continued employment, return to work after > 3 months of sickness absence, or long-term sickness absence (> 3 months) as the outcome variable; (v) at least one of the independent variables contains employer characteristics, including the role of professionals if they interact with the employer; and (vi) the article was written in English. As a consequence these articles are mostly from western countries. In the second phase, the reviewers selected articles for final inclusion based on full-text appraisal. Studies were excluded when both reviewers considered that these did not fulfil the inclusion criteria. Disagreements regarding inclusion were resolved by consensus. If no consensus was reached or in case of doubt, the article was screened by the other authors and discussed to reach consensus. In the third phase, references of included articles were checked for additional relevant articles and we checked for additional recently published articles from the field of economics because of its relatively lengthy publishing process.

### Data Extraction

Two reviewers (JJ, RvO) independently extracted the following characteristics from the included studies: study design, country of the study, scientific discipline, follow-up time, general description of subjects including age and gender, work disability type, outcome measures, employer characteristics and effect sign and size.

### Assessment of Quality

Two reviewers (JJ, RvO) independently assessed the methodological quality of the included studies using nine items [18, 19]. This quality checklist is suitable for assessment of longitudinal observational studies [19]. Table 1 shows the standardized checklist for the quality assessment. Each item was scored positive (+) or negative (−). A negative score was seen as potential bias. The grading of each item was discussed between the reviewers to reach consensus. Based on the nine criteria, the studies were classified as being of high quality when meeting ≥ 8 criteria, medium quality when meeting 6–7 criteria, and low quality when meeting < 6 criteria [11].

**Table 1:** Checklist of methodological quality [18]

Potential biases	Quality assessment criteria
Objective	1. Positive if a clearly stated objective is described
Study population	2. Positive if the main features of the study population are clearly described
	3. Positive if the inclusion and exclusion criteria are clearly described
Outcome	4. Positive if outcome is register-based and if not register-based, the loss to follow up is limited (< 20%)
	5. Positive if a clear definition of employment outcome is given
Determinant	6. Positive if adjusted for health-related confounders (health conditions/severity of the disease/pain level/work ability)
	7. Positive if age (if possible), gender (if possible), education and income are taken into account as confounders
Analysis	8. Positive if appropriate statistical model is used to evaluate data
	9. Positive if effect size of variables was presented or p-value 0.05 was shown or can be calculated

### Evidence Synthesis

A descriptive analysis was undertaken to synthesize the data, which consisted of four stages: grouping, clustering, transforming data and tabulation. Determinants were listed in a stepwise procedure per outcome measure: continued employment, return to work and long-term disability. First, an overview of all determinants that were studied in relation to the work outcomes was created. Determinants referring to the same concept were merged together. For example, the data extraction revealed different aspects of organizational culture, these were merged for evidence grading. Next, determinants were grouped into the following domains: work accommodations, supervisor support, organizational culture and company characteristics. Thirdly, we harmonized the direction of effect sizes. Lastly, we summarized for each domain: (i) the total number of studies reporting on the factor, (i) the number of studies of low, moderate and high quality reporting on the factor, (iii) the scientific disciplines, and (iv) disability types.

### Evidence Grading

**Fig. 1:**
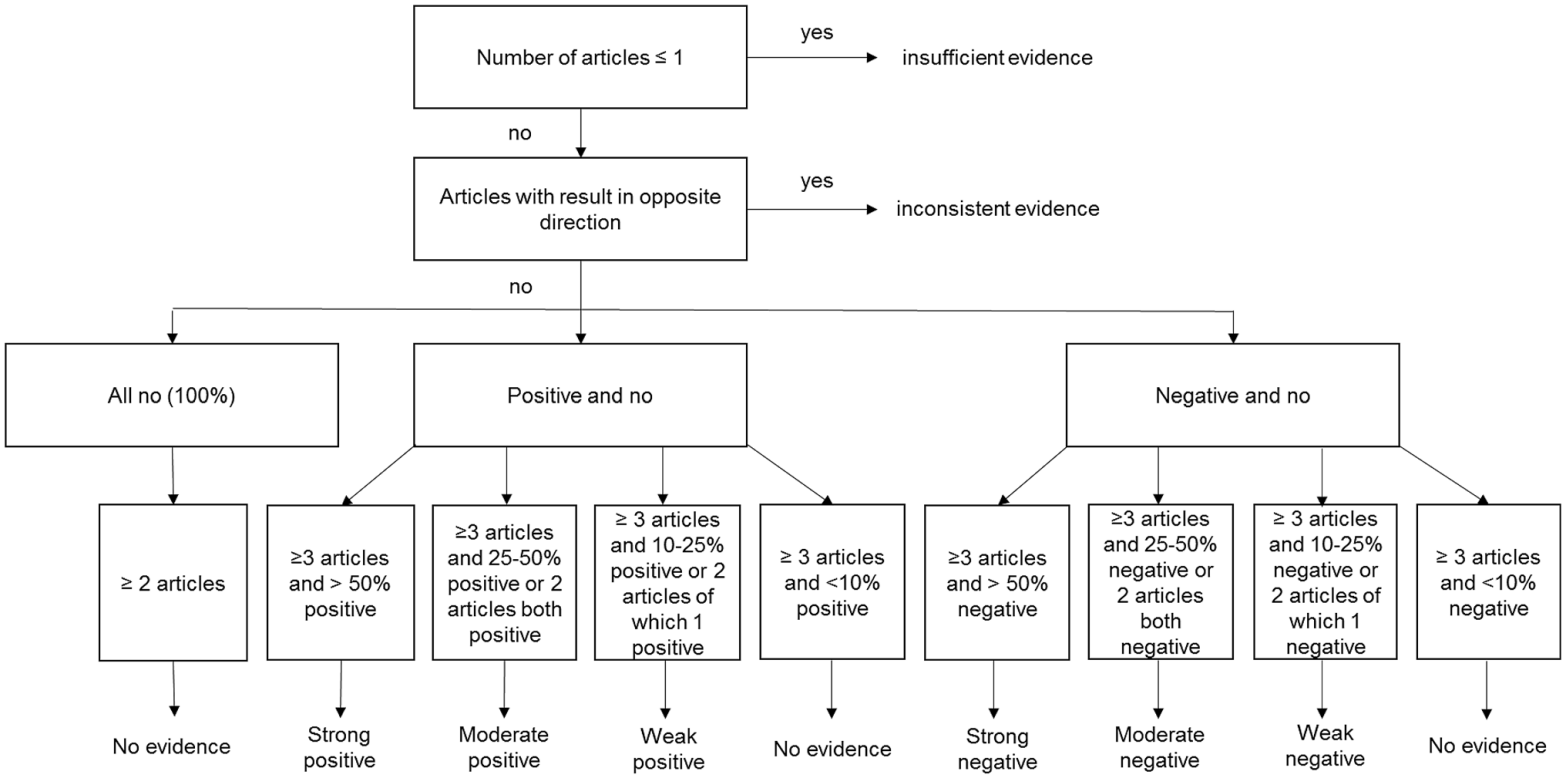
Evidence grading

The level of evidence of the determinants was graded by using the rating system mentioned by de Croon et al. [9]. Ten different evidence levels were determined based on the number of studies and the directions of the effect size. The different evidence grading steps are shown in Fig. 1. Mixed results among the studies with a given outcome does not mean no effect; it means a mixture of negative and positive associations. The level of evidence was established per determinant.

## Results

### Selection of Studies

**Fig. 2:**
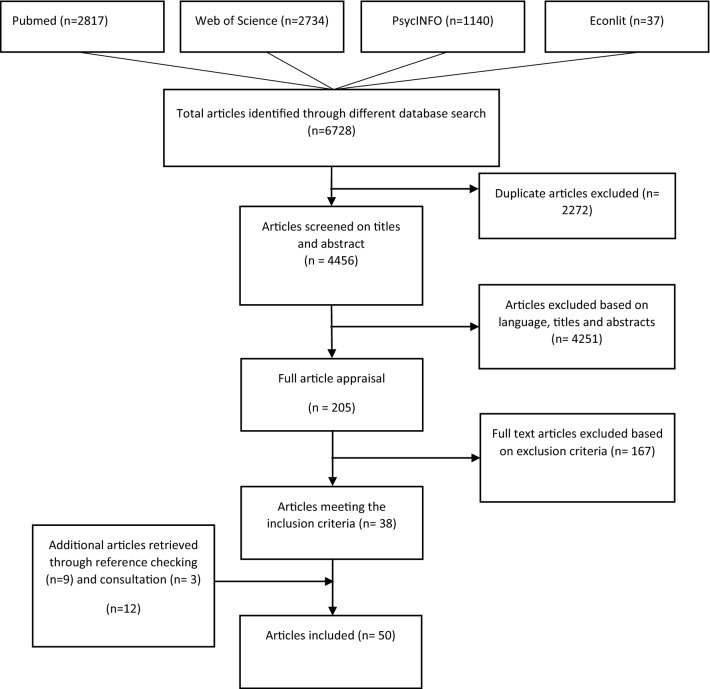
Flow diagram of the selection of studies

The search strategy resulted in 4456 articles, of which 2817 were extracted from Pubmed, 2734 from Web of Science, 1140 from PsycINFO, and 37 from EconLit. After screening on titles and abstracts by the two reviewers, 4251 articles were excluded. A total of 205 articles were selected for further screening. Finally, 38 articles met all inclusion criteria. Further reference checking identified an additional 12 articles, resulting in 50 included articles on 52 individual studies. Figure 2 presents the flow diagram of the selection of studies.

### Study Characteristics

The main characteristics of the included studies are presented in Table 2. Studies varied in work participation outcome measure, scientific disciplines and disability types. Of the 52 studies, 40 investigated determinants in relation to return to work outcomes, 11 studied determinants of continued employment and six studies used long-term disability as a work participation outcome. The economic discipline was represented in 15 studies; the medical discipline in 37 studies. Finally, 28 studies had a specific focus on one specific disability type: mental (n = 11), musculoskeletal (n = 7), cancer (n = 9), diabetes (n = 3), circulatory (n = 2) and nervous (n = 2). The other 20 studies had a broader focus, referred to as work-limiting health conditions. The effect sizes are reported in Table 2 in odds ratios (OR), hazard ratios (HR), rate ratios (RR), propensity score matching (PSM) and marginal effects (ME). The outcome column describes effect sizes of the association between the employer determinant and the outcome, measured at the indicated follow-up period.

**Table 2:** Study characteristics, employer determinants and work outcomes; Study outcome *(S = self-reported, R = register based) **(NR = not reported in the manuscript)

First author, year Country	Sample	Disability type	Scientific discipline	Time to follow-up	Outcome measure	Study outcome*	Employer determinant	Effect size, (95-CI/SE))
Amick, 2017 Canada [56]	Injured Ontario workers on sick-leave Aged 15 + 54.8% male	Musculoskeletal injury	Medical	6 and 12 months	Return to work 6 months	S	Organizational support	OR 1.77 (1.07; 2.93)
					Return to work 12 months			OR 2.07 (1.18; 3.62)
Anema, 2009 Denmark, Germany, Israël, Netherlands, Sweden, United States [33]	Sickness benefit claimants (> 3 months) Age: 18–59 39–74% male (six studies)	Lower back pain	Medical	2 years	Return to work	S and R	Adaptation workplace	HR 0.61 (0.52; 0.71)
							Job redesign	HR 0.57 (0.49; 0.66)
							Working hours adaptation	HR 0.67 (0.57; 0.78)
							Job/vocational training	NR** (insignificant)
							Therapeutic work resumption	HR 0.65 (0.55; 0.78)
Biering, 2015 Denmark [57]	Patients at Aarhus University Hospital treated with PCI on sickness absence > 3 months Age: 25–67 86.2% male	Coronary Heart Disease	Medical	3 and 12 months	Return to work	S and R	Low recognition (rewards)	3 months: OR 2.57 (1.36; 4.86)
								12 months: OR 0.68 (0.33; 1.40)
							Low justice	3 months: OR 1.61 (0.89; 2.92)
								12 months: OR 1.15 (0.57; 2.32)
							Low social community at work	3 months: OR 1.55 (0.82; 2.90)
								12 months: OR 0.94 (0.47; 1.91)
							Low social inclusiveness	3 months: OR 1.14 (0.60; 2.15)
								12 months: OR 0.81 (0.42; 1.57)
Blinder, 2017 United States [20]	Patients treated (stage I–III) at four hospitals and clinics in New York City (> 4 months after treatment) Age 18–64 0% male	Breast cancer	Medical	4 months	Continued employment	S	Employer was accommodating	OR 2.96 (NR, significant)
							Employer size (< 15, ref)	–
							Employer size (15–49)	OR 1.02 (NR, insignificant)
							Employer size (50 and more)	OR 2.65 (NR, significant)
Boot, 2014 Canada [46]	Injured workers on sick-leave having lost-time claims Working age 51% male	Musculoskeletal injury	Medical	12 months	Return to work	S	Positive supervisor response	OR 1.70 (1.17; 2.49)
Bouknight, 2006 United States [25]	Patients with a first primary diagnosis of breast cancer in Detroit area. (> 12 months after diagnosis) Age 30–64 0% male	Breast cancer	Medical	12 and 18 months	Return to work	S	Employer accommodation	12 months: OR 2.2 (1.03; 4.8)
								18 months: OR 2.3 (1.06; 5.1)
Bryngelson, 2012 Sweden [35]	Workers on long-term (> 90 days) sick leave having additional sickness insurance (public sector and manual workers) Age 20–61 17% male	Psychiatric disorder	Medical	3 years	Long-term sickness absence & Newly granted DI	S&R	Workplace-oriented rehabilitation	OR 0.81 (0.68; 0.96)
							Workplace-oriented rehabilitation and no change	OR 0.70 (0.59; 0.83)
							Change of occupation	OR 0.35 (0.27; 0.45)
							Workplace-oriented rehabilitation	OR 1.02 (0.81; 1.27)
Burkhauser, 1999 United States [31]	U.S. workers with a work limiting health condition (> 1 year after sick-leave) Age 21–59 100% male	Work limiting health condition	Economic	up to 17 years	Long-term disability: Applying for DI	S&R	Accommodation (HRS)	HR − 0.60 (SE 0.35)
							Accommodation (SDW)	HR − 0.54 (SE 0.15)
Burkhauser, 1995 United States [24]	U.S. workers with a work limiting health condition (> 1 year after sick-leave) Age 21–59 100% male	Work limiting health condition	Economic	up to 17 years	Continued employment: Job exit	S&R	Accommodation	HR − 1.22 (NR, significant)
Cooper, 2013 United Kingdom [34]	Cancer Patients registered at out-patient departments of hospitals (> 6 months after sick-leave) Aged 18 + 44% male	Breast, Gynecological, Urological, Head and neck cancer	Medical	12 months	Return to work	S	Flexible working allowed	HR 1.70 (1.07; 2.70)
							Company size small (< 60)	NR (insignificant)
							Company size, medium (60–100)	NR (insignificant)
							Company size, large (100 and more)	NR (insignificant)
Daly, 1996 United States [60]	U.S. workers with a work limiting health condition (> 1 year after sick-leave) Age 51–61 57% male	Work limiting health condition	Medical	Up to 17 years	Change employer	S	Number of workers (logarithm)	Men: OR − 0.50 (SE 0.055)
							Number of workers (logarithm)	Women: OR − 0.33 (SE 0.06)
					Stopped working		Number of workers (logarithm)	Men: OR 0.00 (SE 0.052)
							Number of workers (logarithm)	Women: OR 0.03 (SE 0.055)
De Vries, 2015 Netherlands [48]	Sick listed patients at occupational health services in Amsterdam (18 months after sick leave) Age 18–65 55% male	Major depressive disorder	Medical	18 months	Work functioning	S	Supervisor support	NR (insignificant)
Dorland, 2018 Netherlands [44]	Cancer patients who resumed work for at least 12 h/week > 3 months Age 18–65 37% male	Cancer	Medical	n/a	Work functioning	S	Social support supervisor	ME 0.71 (0.29; 1.13)
Ekberg, 2015 Sweden [58]	Patients on sick leave for at least 3 months in Östergötland Age 18–65 67% male	Common Mental Disorders	Medical	3 to 12 months	Return to work	S & R	Organizational culture (justice)	NR (insignificant)
Engström, 2007 Sweden [68]	Sick registered individuals (1–3 years after sick leave) in the county of Värmland. Working age population 23.5% male	stress-related psychiatric disorders	Medical	2 years	Return to work (partial)	R	County, health	OR 0.37 (NR, significant)
							Private	OR 0.64 (NR, insignificant)
							Municipality, education	OR 0.80 (NR, insignificant)
							Municipality, other	OR 0.83 (NR, insignificant)
							Municipality, health (elderly care)	OR 0.84 (NR, insignificant)
							County, other	OR 0.95 (NR, insignificant)
							Public, other (ref.)	–
					Return to work (full)		County, health	OR 0.42 (NR, insignificant)
							County, other	OR 0.73 (NR, insignificant)
							Private	OR 0.74 (NR, insignificant)
							Municipality, health (elderly care)	OR 0.89 (NR, insignificant)
							Municipality, education	OR 0.92 (NR, insignificant)
							Municipality, other	OR 1.09 (NR, insignificant)
							Public, other (ref.)	–
Ervasti, 2016 Finland, UK and France [49]	Employees with diabetes on sick-leave for at least 1 year. Working age population 28%, 70%, 76% male	Diabetes	Medical	1 to 5 years	Absence duration	S&R	Low supervisor support	Finland; Women RR 1.09 (0.74; 1.61)
							Low supervisor support	Finland; Men RR 1.23 (0.67; 2.65))
					Absence duration		Low supervisor support	UK; Women RR 1.33 (0.65; 2.74)
							Low supervisor support	UK; Men RR 1.27 (0.60; 2.67)
					Return to work		Low supervisor support	France; Women RR 1.82 (0.70; 4.73)
							Low supervisor support	France; Men RR 0.98 (0.43; 2.23)
Everhardt, 2011 Netherlands [26]	Workers on long-term sick leave (> 9 months) Working age population 55% male	Work limiting health condition	Economic	18 months	Return to work	S	Accommodation (employer)	HR 1.89 (NR, significant)
							Accommodation (occupational health service)	HR 1.48 (NR, significant)
							Accommodation (other agency)	HR 0.76 (NR, significant)
							Return to work-plan	HR 1.25 (NR, significant)
Faucett, 2000 United States [32]	Patients in Santa Clara County (> 18 months after sick leave) Working age population 24% male	Carpal tunnel syndrome	Medical	18 months	Active employment	S	Supervisor support	NR (insignificant)
							Employer size <250	OR 13.61 (1.24; 149.80)
							Work accommodation (work change)	OR 10.30 (1.12; 94.59)
					Job change (any)		Supervisor support	HR 0.71 (0.29; 1.78)
							Size	HR 1.64 (0.49; 5.46)
							Work accommodation (work change)	HR 1.13 (0.33; 3.88)
Franche, 2007 Canada [27]	Sick listed Ontario workers (> 6 months) at firms with workers’ compensation coverage Aged 15 + 53.4% male	Musculoskeletal	Medical	6 months	Return to work	S&R	Work accomodation offer rejected	HR 0.53 (0.39; 0.72)
							No work accomodation offered	HR 0.46 (0.38; 0.57)
							No contact between HCP and the workplace	HR 1.24 (NR insignificant)
							No advice from HCP to the workplace	HR 0.56 (NR significant)
							Ergonomic worksite visits	HR 1.44 (NR significant)
							Return to work coordinator	HR 0.84 (NR insignificant)
Frölich, 2004 Sweden [36]	Sicklisted workers in Western Sweden (> 8 months) Working-age population 40% male	Work limiting health condition	Economic	8–42 months	Return to work	R	No rehabilitation (reference)	–
							Passive rehabilitation	PSM − 12.0 (NR, significant)
							Workplace rehabilitation (vocational work training)	NR (insignificant)
							Educational rehabilitation	PSM − 18.7 (NR, significant)
							Medical rehabilitation	PSM − 7.8 (NR, significant)
							Social rehabilitation	NR (insignificant)
Gordon, 2014 Australia [62]	Newly-diagnosed patients in Queensland (12 months after sick-leave) Age 45–64 67% male	Colorectal cancer	Medical	12 months	Time to work resumption	S	Employer size < 20 (ref.)	–
							Employer size (20–100)	OR 1.66 (1.09; 2.53)
							Employer size (> 100)	OR 1.47 (0.83; 2.60)
Hannerz, 2012 Denmark [61]	Previously employed stroke-patients Age 21–57 60.4% male	Stroke	Medical	2 years	Return to work	R	Employer size < 10 (ref. 250 +)	OR 0.83 (0.73; 0.95)
							Employer size 10–49	OR 0.87 (0.77; 0.98)
							Employer size 50–249	OR 0.90 (0.80; 1.01)
Haveraaen, 2014 Norway [50]	Sick-listed employees who participated in return to work services NR 23.9% male	Work limiting health condition	Medical	3 months	Return to work	S&R	Supervisor support (high)	OR 3.94 (1.57; 7.31)
Hill, 2016 United States [21]	Newly disabled workers Aged 51 + 41% male	Work limiting health condition	Economic	2 and 4 years	Continued employment	S	Accommodation	2 years: ME 0.171 (SE 0.033)
							Accommodation—Work change	2 years: ME 0.273 (NR significant)
					Continued employment		Accommodation—Changes to time	2 years: ME 0.162 (NR significant)
							Accommodation—Equipment/assistance	2 years: ME 0.118 (NR significant)
					Continued employment		Accommodation—Other	2 years: ME 0.105 (NR significant)
							Accommodation	4 years: ME 0.045 (SE 0.037)
					Receiving DI/ Applying for DI		Accommodation	4 years: ME 0.017 (SE 0.032)
							Accommodation	4 years: ME − 0.037 (SE 0.035)
Hogelund, 2006 Denmark [37]	Long-term sick-listed employees Working-age population 44% male	Work limiting health condition	Economic	Up to 7 years	Return to work	S&R	Case management interview	HR 1.69 (SE 0.943)
					Return to work for pre-sick leave employer		Case management interview	HR 2.77 (SE 1.095)
					Return to work for new employer:		Case management interview	HR − 0.73 (SE 1.694)
	Employees who did not participate in vocational rehabilitation				Return to work		Case management interview	HR 2.37 (SE 1.013)
					Return to work for pre-sick leave employer		Case management interview	HR 3.94 (SE 1.155)
					Return to work for new employer		Case management interview	HR − 1.94 (SE 1.85)
					Return to work		Sector	NR (insignificant)
Hogelund, 2014 Denmark [22]	Long-term sick-listed employees Working age population 36% male	Work limiting health condition	Economic	Up to 28 months	Ending employment	S&R	Workplace accommodations, current employer	HR − 0.527 (SE 0.267)
							Reduced working hours, current employer	HR − 0.476 (SE 0.314)
							New job, current employer	HR 0.021 (SE 0.424)
							Light duties, current employer	HR − 0.273 (SE 0.463)
							Adaptations, current employer	HR − 0.471 (SE 0.481)
							New employer	HR 0.592 (SE 0.254)
							Company size	NR (insignificant)
							Public sector company	HR − 0.329 (SE 0.208)
Janssen, 2003 Netherlands [51]	Long-term sick-listed employee Age 19–60 71% male	Work limiting health condition	Medical	4 months	Full return to work	S	Supervisor support	OR 1.40 (1.08; 1.83)
					Return to work with adjustments		Supervisor support	OR 1.17 (0.93; 1.48)
					Full return to work versus return to work with adjustments		Supervisor support	OR 1.18 (0.92; 1.51)
Katz, 2005 United States [52]	Patients in the state of Maine Aged 18 + 42% male	Carpal tunnel syndrome	Medical	6 and 12 months	Work absence	S	Social support of supervisors	NR (insignificant)
							Number of employees	Return to work with adjustments: NR (insignificant)
							Organizational policies and practices (less supportive)	12 months: OR 2.94 (1.18; 7.34)
							Organizational policies and practices (less supportive)	6 months full return to work versus return to work with adjustments: NR (insignificant)
Kools, 2019 Netherlands [39]	Sick-listed employees assigned to a large private workplace reintegration provider Working age population 53% male	Work limiting health condition	Economic	1 and 2 year	Return to work 12 months	R	Graded return to work (first year)	ME 0.13 (SE 0.122)
					Return to work 24 months		Graded return to work (first year)	ME 0.08 (SE 0.109)
					Return to work 12 months		Graded return to work (first semester)	ME 0.38 (SE 0.125)
					Return to work 24 months		Graded return to work (first semester)	ME 0.07 (SE 0.104)
Lindbohm, 2014 Denmark [45]	Breast cancer patients. The data is from a cross-sectional dataset and the analyses is longitudinal retrospective Age 25–57 0% male	Breast cancer	Medical	1–8 years	Non-employed (excl. early retirement)	S&R	Moderate support from the supervisor (ref. high)	OR 0.95 (0.43; 2.08)
							Weak support from the supervisor (ref. high)	OR 2.51 (1.10; 5.72)
Lund, 2006 Denmark [63]	Sick listed employees Working age population 50% male	Work limiting health condition	Medical	1 year	Return to work	S&R	Private	HR 1.21 (1.04; 1.41)
							< 20(ref.)	–
							20–100 (< 20 baseline)	HR 0.86 (0.74; 1.00)
							> 100 (< 20 baseline)	HR 0.86 (0.73; 1.00)
Markussen, 2011 Norway [64]	Sick-listed employees certified by a physician Age 30–60 NR	Work limiting health condition	Economic	1 year	Return to work (minor disease)	R	Firm with less than 20 employees	HR − 0.02 (NR significant)
							Mining	HR − 0.14 (NR)
							Transportation	HR − 0.10 (NR)
							Agriculture	HR − 0.05 (NR)
							Other	HR − 0.04 (NR)
							Construction	HR − 0.04 (NR)
							Health	HR − 0.03 (NR)
							Public administration	HR − 0.03 (NR)
							Wholesale and retail trade	HR − 0.03 (NR)
							Education	HR − 0.03 (NR)
							Recreation	HR − 0.02 (NR)
							Professional and administrative services	HR − 0.02 (NR)
							Accomodation and restaurants	HR − 0.02 (NR)
							Information and communication	HR − 0.01 (NR)
							Financial and insurance	HR − 0.01 (NR)
							Manufacturing	HR − 0.01 (NR)
							Real estate	HR − 0.00 (NR)
							Utilities	HR 0.01 (NR)
					Return to work (major disease)		Firm with less than 20 employees	HR − 0.12 (significant)
							Transportation	HR − 0.13 (NR)
							Real estate	HR − 0.12 (NR)
							Mining	HR − 0.11 (NR)
							Wholesale and retail trade	HR − 0.10 (NR)
							Education	HR − 0.10 (NR)
							Professional and administrative services	HR − 0.10 (NR)
							Public administration	HR − 0.09 (NR)
							Financial and insurance	HR − 0.08 (NR)
							Agriculture	HR − 0.08 (NR)
							Other	HR − 0.05 (NR)
							Information and communication	HR − 0.05 (NR)
							Manufacturing	HR − 0.04 (NR)
							Recreation	HR − 0.03 (NR)
							Accomodation and restaurants	HR − 0.03 (NR)
							Health	HR − 0.02 (NR)
							Utilities	HR − 0.00 (NR)
							Construction	HR 0.07 (NR)
Markussen, 2012 Norway [42]	Long-term sick-listed employees handled by the family doctor. Working age population 44% male	Work limiting health condition	Economic	24 months	Employment	R	Graded return to work	ME 0.21 (SE 0.03)
					Days on social security		Graded return to work	ME − 102.30 (SE 8.2)
					Absense duration days		Graded return to work	ME − 58.80 (SE 8.0)
Markussen, 2014 Norway [43]	Entrants into the temporary disability insurance program Age 18–57 46% male	Work limiting health condition	Economic	12 months	Continued employment	R	Placement in regular firms, with or without individual support	ME 11.66 (SE 5.74)
					Long-term disability		Placement in regular firms, with or without individual support	ME − 12.94 (SE 7.26)
Markussen, 2018 Norway [38]	Long-term sick-listed employees (after ± 6 months) certified by a physician Age 18–66 42% male	Work limiting health condition	Economic	12 months	Return to work (days)	R	Compulsory dialog meetings—high/mixed intensity	ME − 20.30 (NR, significant)
							Compulsory dialog meetings—high/low intensity	ME − 19.00 (NR, significant)
McLaren, 2017 United States [28]	Workers’ compensation data from private and public firms	Work limiting health condition	Economic	5 years	Return to work	S&R	Return to work program	HR 1.38 ((NR, significant)
							Modified work	HR 1.27 (NR, significant)
							Different job (same firm)	HR 0.70 (NR, significant)
							Scheduling accomodations	HR 1.22 (NR, insignificant)
							Modified equipment	HR 1.50 (NR, significant)
Mehnert, 2013 Germany [29]	Patients from cancer rehabilitation facilities Age 18–60 14.3% male	Cancer (mainly breast cancer and gynecological cancer)	Medical	12 months	Reemployment	S	Perceived employer accommodation	OR 1.93 (1.41; 2.65)
					Time to RTW		Perceived employer accommodation	HR 1.18 (1.06; 1.32)
Muijzer, 2011 Netherlands [53]	Employees applying for disability benefits after 2 years of sickness absence Working age population 43% male	Physical or Mental	Medical	2 year	No return to work (full/partial)	S	Relationship employer/employee (poor)	OR 14.59 (3.29; 64.71)
							Conflict with supervisor	NR (insignificant)
Netterstrom, 2015 Denmark [54]	Patients on sick leave Working age population 19.7% male	Work-Related Common Mental Disorders	Medical	1 year & 3 years	Return to work	S&R	Low support from leader	1 year NR (significant)
							Low support from leader	3 years NR (insignificant)
Neumark, 2015 United States [23]	Patients in eight centers in Virginia Age 21–64 0% male	Breast cancer	Economic	9 months	Employment	S	Any accommodation	ME 0.019 (SE 0.05)
							Helper at work	ME 0.024 (SE 0.028)
							Shorter day	ME − 0.030 (SE 0.029)
							Allowed schedule change	ME − 0.008 (SE 0.044)
							Allowed more breaks	ME 0.037 (SE 0.034)
							Special transportation	ME − 0.126 (SE 0.085)
							Job change	ME 0.008 (SE 0.039)
							Help learning new skills	ME 0.026 (SE 0.046)
							Special equipment	ME 0.062 (SE 0.044)
							Assistance with rehabilitative services	ME 0.121 (SE 0.055)
Nielsen, 2012 Denmark [65]	Employees on sick leave in Copenhagen Working age population 20.5% male	Mental health problems	Medical	52 weeks	Return to work	S&R	Size > 250	NR (insignificant)
							Municipal	0.62 (0.41; 0.94)
							Private (ref. governmental)	0.65 (0.44; 0.96)
							Governmental (ref)	-
Nieuwenhuijsen, 2004 Netherlands [40]	Patients on sick leave at nine occupational health service center and their supervisors Working-age population 42% male	Mental health problems	Medical	1 year	Return to work (full)	S&R	Communication with employee	HR 1.7 (1.0; 2.8)
							Promoting gradual return to work	HR 0.8 (0.4; 1.5)
							Consulting with professionals	HR 0.6 (0.4; 1.0)
					Return to work (partial)		Communication with employee	HR 1.3 (0.8; 2.0)
							Promoting gradual return to work	HR 0.9 (0.5; 1.5)
							Consulting with professionals	HR 0.7 (0.5; 1.2)
Nieuwenhuijsen, 2006 Netherlands [55]	Sick listed workers from nine occupational health services Working age population 40% male	Common mental disorders	Medical	12 months	Full return to work	S&R	Supervisory support	HR 1.1 (NR, insignificant)
Prang, 2016 Australia [66]	Claimants (non-federal government) Age 15–70 44% male	Mental health condition (work related)	Medical	2 years	Return to work	R	Workplace size—small (ref. Government)	HR 0.81 (NR, significant)
							Workplace size—medium	HR 0.97 (NR, significant)
							Workplace size—large	HR 1.15 (NR, significant)
							Scientific and technical services	HR 0.72 (0.62; 0.92)
							Education	HR 0.74 (0.68; 0.80)
							Information and communication	HR 0.75 (0.62; 0.92)
							Financial and insurance	HR 0.76 (0.63; 0.91)
							Public administration	HR 0.77 (0.71; 0.83)
							Manufacturing	HR 0.79 (0.71; 0.87)
							Wholesale trade	HR 0.80 (0.69; 0.91)
							Agriculture	HR 0.81 (0.62; 1.07)
							Retail trade	HR 0.81 (0.71; 0.93)
							Real estate	HR 0.83 (0.68; 1.01)
							Construction	HR 0.87 (0.73; 1.03
							Administrative services	HR 0.87 (0.74; 1.03)
							Utilities	HR 0.88 (0.67; 1.15)
							Accomodation and food services	HR 0.89 (0.75; 1.05)
							Other services	HR 0.89 (0.78; 1.02)
							Mining	HR 0.92 (0.47; 1.77)
							Recreation	HR 0.92 (0.78; 1.10)
							Health (ref.)	–
							Transportation	HR 1.24 (1.11; 1.38)
Post, 2005 Netherlands [47]	Employees on sickness absence Age 18–63 50% male	Work limiting health condition	Medical	10 months	Return to work	S	Supervisor support (low)	RR 1.00–
							Supervisor support (high)	RR 1.23 (1.02; 1.49)
							Health care and welfare services	RR 1.00–
							Industry	RR 1.20 (0.96; 1.52)
							Trade	RR 1.07 (0.67; 1.70)
							Culture, recreation and other services	RR 0.89 (0.60; 1.34)
							Construction	RR 0.85 (0.62; 1.18)
							Other	RR 0.83 (0.48; 1.43)
							Public administration	RR 0.78 (0.57; 1.05)
							Transport	RR 0.78 (0.52; 1.16)
							Financial and commercial services	RR 0.74 (0.49; 1.13)
							Education	RR 0.46 (0.35; 0.61)
							Company size 1–9	RR 0.64 (0.39; 1.05)
							Company size 10–99	RR 0.79 (0.65; 0.94)
							Company size > 100	RR 1.00–
Schneider, 2016 Germany [41]	Sickness fund claimants Working age population 52% Male	Work limiting health condition	Economic	17 months	Return to work		Size < 50 (ref.)	–
							Size 50–249	HR 1.02 (SE 0.5161)
							Size > 250	HR 1.07 (SE 0.0013)
							Graded return-to-work program	Sickness absence < 120 days HR < 1.0 (NR, significant)
							Graded return-to-work program	Sickness absence > 120 days HR > 1.0 (NR, significant)
Schroër, 2005 Netherlands [59]	Employees on sick leave. Working age population 70% male	Work limiting health condition	Medical	15 months	Return to work	S	Private (ref. public)	OR 2.02 (significant)
							Size < 800 employees	OR 0.89 (0.41; 1.95)
							Job/employee oriented culture	OR 0.63 (0.31; 1.28)
							Process/result-oriented culture	OR 0.97 (0.45; 2.12)
							Open/closed culture	OR 1.82 (0.92; 3.36)
Smith, 2014 Australia [67]	Claimants receiving wage replacement. Working age population 58% male	Mental and Musculoskeletal	Medical	24 months	Days away from work	R	Small	Mental: HR 0.13 (SE 0.08)
							Medium (reference)	–
							Large/Government	Mental: HR − 0.23 (SE 0.06)
							Small	Musculoskeletal: HR 0.43 (SE 0.04)
							Medium (reference)	–
							Large/Government	Musculoskeletal: HR − 0.21 (SE 0.04)
							Healthcare	Musculoskeletal: HR − 0.27 (NR)
							Education	Musculoskeletal: HR − 0.26 (NR)
							Public administration	Musculoskeletal: HR − 0.17 (NR)
							Retail trade	Musculoskeletal: HR − 0.05 (NR)
							Other	Musculoskeletal: HR − 0.03 (NR)
							Wholesale trade	Musculoskeletal: HR 0.00 (NR)
							Transport	Musculoskeletal: HR 0.04 (NR)
							Agriculture	Musculoskeletal: HR 0.06 (NR)
							Construction	Musculoskeletal: HR 0.22 (NR)
							Manufacturing (reference)	–
Turner, 2008 United States [30]	Claimants (who receive some wage replacement) Working age population 68% male	Back injury (work related)	Medical	12 months	Work disability	S& R	Job accommodation not offered	OR 1.91 (1.31; 2.76)
							Employer size	NR (insignificant)
							Mining (ref. trade & transportation)	OR 1.02 (0.42; 2.48)
							Construction	OR 1.88 (1.12; 3.17)
							Manufacturing	OR 1.98 (1.04; 3.77)
							Management	OR 1.08 (0.62; 1.89)
							Education/health	OR 0.92 (0.49; 1.74)
							Hospitality	OR 1.05 (0.58; 1.91)
Veenstra, 2018 United States [69]	Patients with stage III colorectal cancer Age > 18 years 57% male	Colorectal cancer	Medical	12 months	Job retention	S	Employer-based health insurance	HR 2.97 (1.56; 6.01)
							Paid sick leave	HR 2.93 (1.23; 6.98)
							Extended sick leave	HR 1.41 (0.61; 2.12)
							Unpaid time off	HR 0.79 (0.44; 1.40)
							Disability benefits	HR 0.55 (0.27; 1.14)

*(S = self-reported, R = register based)

**(NR = not reported)

***The data is from a cross-sectional dataset and the analysis is longitudinal retrospective

### Quality Assessment

The results of the quality assessment are presented in Table 3. In total, 39 out of 50 articles (78%) were graded to be of high quality, whereas the other 11 articles (22%) were graded as medium quality. No low quality articles were found.

**Table 3:** Results quality assessment

Key	Publication	1	2	3	4	5	6	7	8	9	Total score		Quality
1	Amick 2017 [56]	+	+	+	−	+	+	−	+	+		7/9	MQ
2	Anema 2009 [33]	+	+	+	+	+	+	+	+	+		9/9	HQ
3	Biering 2015 [57]	+	+	+	+	+	+	+	+	+		9/9	HQ
4	Blinder 2017 [20]	+	+	+	+	+	+	+	+	+		9/9	HQ
5	Boot 2014 [46]	+	+	+	−	+	+	−	+	+		7/9	MQ
6	Bouknight 2006 [25]	+	+	+	+	+	+	+	+	+		9/9	HQ
7	Bryngelson 2012 [35]	+	+	+	+	+	+	+	+	+		9/9	HQ
8	Burkhauser 1995 [24]	+	+	+	+	+	+	+	+	+		9/9	HQ
9	Burkhauser 1999 [31]	+	+	+	+	+	+	+	+	+		9/9	HQ
10	Cooper 2013 [34]	+	+	+	+	+	−	−	+	+		7/9	MQ
11	Daly 1996 [60]	+	+	+	+	+	+	+	+	+		9/9	HQ
12	De Vries 2015 [48]	+	+	+	−	+	−	−	+	+		6/9	MQ
13	Dorland 2018 [44]	+	+	+	−	+	+	+	+	+		8/9	HQ
14	Ekberg 2015 [58]	+	+	+	+	+	−	−	+	−		6/9	MQ
15	Engström 2007 [68]	+	+	+	+	+	+	+	+	+		9/9	HQ
16	Ervasti 2016 [49]	+	+	+	+	+	+	+	+	+		9/9	HQ
17	Everhardt 2011 [26]	+	−	+	−	+	+	+	+	+		7/9	MQ
18	Faucett 2000 [32]	+	+	+	+	+	+	−	+	+		8/9	HQ
19	Franche 2007 [27]	+	+	+	+	+	+	+	+	+		9/9	HQ
20	Fröhlich 2004 [36]	+	+	+	+	+	+	+	+	+		9/9	HQ
21	Gordon 2014 [62]	+	+	+	−	+	−	−	+	+		6/9	MQ
22	Hannerz 2012 [61]	+	+	+	+	+	+	+	+	+		9/9	HQ
23	Haveraaen 2014 [50]	+	+	+	+	+	+	+	+	+		9/9	HQ
24	Hill 2016 [21]	+	+	+	+	+	+	+	+	+		9/9	HQ
25	Hogelund 2006 [37]	+	+	+	+	+	+	+	+	+		9/9	HQ
26	Hogelund 2014 [22]	+	+	+	+	+	+	+	+	+		9/9	HQ
27	Janssen 2003 [51]	+	+	+	+	+	−	−	+	+		7/9	MQ
28	Katz 2005 [52]	+	+	+	+	+	+	−	+	−		7/9	MQ
29	Kools 2019 [39]	+	+	+	+	+	+	+	+	+		9/9	HQ
30	Lindbohm 2014 [45]	+	+	+	+	+	+	+	+	+		9/9	HQ
31	Lund 2006 [63]	+	−	+	+	+	+	+	+	+		8/9	HQ
32	Markussen 2012 [42]	+	+	+	+	+	+	+	+	+		9/9	HQ
33	Markussen 2011 [64]	+	+	+	+	+	−	+	+	+		8/9	HQ
34	Markussen 2014 [43]	+	+	+	+	+	+	+	+	+		9/9	HQ
35	Markussen 2018 [38]	+	+	+	+	+	+	+	+	+		9/9	HQ
36	McLaren 2017 [28]	+	+	+	+	+	+	+	+	+		9/9	HQ
37	Mehnert 2013 [29]	+	+	+	−	+	+	+	+	+		8/9	HQ
38	Muijzer 2011 [53]	+	−	+	+	+	−	+	+	+		7/9	MQ
39	Netterstrom 2015 [54]	+	+	+	+	+	−	+	+	−		7/9	MQ
40	Neumark 2015 [23]	+	+	+	+	+	+	+	+	+		9/9	HQ
41	Nielsen 2012 [65]	+	+	+	+	+	+	−	+	+		8/9	HQ
42	Nieuwenhuijsen 2004 [40]	+	+	+	+	+	+	+	+	+		9/9	HQ
43	Nieuwenhuijsen 2006 [55]	+	+	+	+	+	+	+	+	+		9/9	HQ
44	Post 2005 [47]	+	+	+	+	+	−	+	+	+		8/9	HQ
45	Prang 2016 [66]	+	+	+	+	+	+	+	+	+		9/9	HQ
46	Schneider 2016 [41]	+	+	+	+	+	+	+	+	+		9/9	HQ
47	Schröer 2005 [59]	+	+	+	+	+	+	+	+	+		9/9	HQ
48	Smith 2014 [67]	+	+	+	+	+	+	+	+	+		9/9	HQ
49	Turner 2008 [30]	+	+	+	+	+	+	+	+	+		9/9	HQ
50	Veenstra 2018 [69]	+	+	+	−	+	+	+	+	+		8/9	HQ

### Employer Determinants

In total, we found 14 determinants that could be clustered in the following four domains: work accommodations, social support, organizational culture and company characteristics (see Table 4).

**Table 4:** Overview of evidence grading per determinant

Domain	Determinants	Work participation outcome	Evidence	Nr. of studies	Ref. nr	Quality assessment	Scientific discipline	Disability type
Work accommodation	1. Any accommodation	Continued employment	Strong +	5	[20–24]	High (n = 5)	Economic (n = 4) Medical (n = 1)	Work-limiting health condition (n = 3) Cancer (n = 2)
		Return to work	Strong +	5	[25–29]	High (n = 4) Medium (n = 1)	Economic (n = 2) Medical (n = 3)	Work-limiting health condition (n = 2) Cancer (n = 2) Musculoskeletal (n = 1)
		Long-term disability	Moderate +	3	[21, 30, 31]	High (n = 3)	Economic (n = 2) Medical (n = 1)	Work-limiting health condition (n = 2) Musculoskeletal (n = 1)
	2. Work change	Continued employment	Moderate +	4	[21–23, 32]	High (n = 4)	Economic (n = 3) Medical (n = 1)	Work-limiting health condition (n = 2) Cancer (n = 1) Nervous (n = 1)
		Return to work	Inconsistent	3	[28, 33, 35]	High (n = 3)	Economic (n = 1) Medical (n = 2)	Work-limiting health condition (n = 1) Musculoskeletal (n = 1) Mental (n = 1)
	3. Employer change	Continued employment	Inconsistent	1	[22, 43]	High (n = 2)	Economic (n = 2)	Work-limiting health condition (n = 2)
		Long-term disability	Insufficient	1	[43]	High (n = 1)	Economic (n = 1)	Work-limiting health condition (n = 1)
	4. Time	Continued employment	Moderate +	3	[21–23]	High (n = 3)	Economic (n = 3)	Work-limiting health condition (n = 2) Cancer (n = 1)
		Return to work	Strong +	3	[28, 33, 34]	High (n = 2) Medium (n = 1)	Medical (n = 2) Economic (n = 1)	Work-limiting health condition (n = 1) Cancer (n = 1) Musculoskeletal (n = 1)
	5. Workplace intervention	Return to work	Strong +	6	[26, 33, 35–38]	High (n = 5) Medium (n = 1)	Economic (n = 4) Medical (n = 2)	Work-limiting health condition (n = 4) Musculoskeletal (n = 1) Mental (n = 1)
		Long-term disability	Insufficient	1	[35]	High (n = 1)	Medical (n = 1)	Mental (n = 1)
	6. Graded return to work	Continued employment	Insufficient	1	[42]	High (n = 1)	Economic (n = 1)	Work-limiting health condition (n = 1)
		Return to work	Weak +	4	[39–42]	High (n = 4)	Economic (n = 3) Medical (n = 1)	Work-limiting health condition (n = 3) Mental (n = 1)
		Long-term disability	Insufficient	1	[42]	High (n = 1)	Economic (n = 1)	Work-limiting health condition (n = 1)
	7. Professional assistance at work	Continued employment	Insufficient	1	[23]	High (n = 1)	Economic (n = 1)	Cancer (n = 1)
		Return to work	Insufficient	1	[27]	High (n = 1)	Medical (n = 1)	Musculoskeletal (n = 1)
	8. Professional assistance outside work	Continued employment	Insufficient	1	[23]	High (n = 1)	Economic (n = 1)	Cancer (n = 1)
		Return to work	Inconsistent	3	[26, 27, 40]	High (n = 2) Medium (n = 1)	Economic (n = 1) Medical (n = 2)	Work-limiting health condition (n = 1) Musculoskeletal (n = 1) Mental (n = 1)
	9. Equipment assistance	Continued employment	Weak +	3	[21–23]	High (n = 3)	Economic (n = 3)	Work-limiting health condition (n = 2) Cancer (n = 1)
		Return to work	Strong +	3	[27, 28, 33]	High (n = 3)	Economic (n = 1) Medical (n = 2)	Work-limiting health condition (n = 1) Musculoskeletal (n = 2)
	10. Employer provided health/ sick leave /disability insurance	Continued employment	Moderate +	2	[20, 69]	High (n = 2)	Medical (n = 2)	Cancer (n = 2)
Social support	11. Supervisor support	Continued employment	Weak +	2	[32, 45]	High (n = 2)	Medical (n = 2)	Cancer (n = 1) Nervous (n = 1)
		Return to work	Moderate +	14	[40, 44, 46–55]	High (n = 8) Medium (n = 6)	Medical (n = 14)	Work-limiting health condition (n = 3) Musculoskeletal (n = 2) Mental (n = 5) Diabetes (n = 3) Nervous (n = 1) Cancer (n=1)
Organizational culture	12. Organizational culture	Return to work	Weak +	5	[52, 56–59]	High (n = 2) Medium (n = 3)	Medical (n = 5)	Work-limiting health condition (n = 1) Musculoskeletal (n = 1) Mental (n = 1) Circulatory (n = 1) Nervous (n = 1)
Company characteristics	13. Company size	Continued employment/	Inconsistent	47	[20, 22, 32, 60]	High (n = 4)	Economic (n = 1) Medical (n = 3)	Work-limiting health condition (n = 2) Cancer (n = 1) Nervous (n = 1)
		Return to work	Inconsistent	12	[34, 41, 47, 52, 59, 61–67]	High (n = 9) Medium (n = 3)	Economic (n = 2) Medical (n = 10)	Work-limiting health condition (n = 5) Musculoskeletal disorder (n = 1) Cancer (n = 2) Mental (n = 3) Nervous (n = 1) Circulatory (n=1)
		Long-term disability	Insufficient	1	[30]	High (n = 1)	Medical (n = 1)	Musculoskeletal disorder (n = 1)
	14. Sector	Continued employment	Insufficient	1	[22]	High (n = 1)	Economic (n = 1)	Work-limiting health condition (n = 1)
		Return to work	Inconsistent	9	[37, 47, 59, 63–68]	High (n = 9)	Economic (n = 2) Medical (n = 7)	Work-limiting health condition (n = 5) Musculoskeletal (n = 1) Mental (n = 4)

#### Work Accommodations

Work accommodation, defined in studies as having an accommodating employer or offered accommodations, was found to be related to continued employment [20–24] and faster return to work [25–29]. Moderate evidence was found for this determinant related to reduced long-term disability [21, 30, 31].

Nine different types of work accommodations were studied: work change, employer change, work-time change, workplace interventions, professional assistance at the workplace, professional assistance outside the workplace, graded return to work, equipment assistance, and employer provided health/disability insurance. There was moderate evidence that work change, defined as change in job tasks and change in work, was positively associated with continued employment [21–23, 32]. Change in work time and flexibility in time scheduling was strongly positively associated with return to work [28, 33, 34]. There was less evidence pointing at effects of change in work time on continued employment [21–23] and employer change [22, 43]. Workplace programs on guidance and support such as vocational work training, case management interviews and occupational health services was strongly positively associated with return to work [26, 33, 35–38]. In addition, we found weak evidence for a positive association between graded return to work programs and return to work [39–42], and a weak positive association between equipment assistance and continued employment [21–23]. Strong evidence was found between equipment assistance and return to work [27, 28, 33]. For return to work, we found inconsistent evidence for the following determinants: work change [28, 33, 35] and professional assistance outside the workplace [26, 27, 40].

For some determinants and outcomes, we did not find sufficient studies to assess the evidence. For continued employment, this was the case for the following determinants: graded return to work [42], professional assistance at work [23] and professional assistance outside the workplace [23]. For return to work, this concerns the determinant professional assistance at the workplace [27]. For long-term disability, this concerns the determinants employer change [43], workplace interventions [35], graded return to work [42].

#### Social Support

Social support, includes measures of the relationship between the supervisor and the worker, measures of supervisor support and measures relating to the presence of conflicts between supervisor and worker. Weak evidence was found for a positive association with continued employment [32, 45]. For return to work moderate evidence was found for this association [40, 44, 46–55]. No studies were found for long-term disability.

#### Organizational Culture

Determinants related to organizational culture, like injustice, open versus closed culture, less supportive policies and practices were only studied in relation to return to work. The overall evidence for these determinants was weak [52, 56–59].

#### Company Characteristics

Two company characteristics identified in the included studies of interest were company size and sector. Inconsistent evidence was found for the associations between company size and continued employment [20, 22, 32, 60] and return to work [34, 41, 47, 52, 59, 61–67]. Insufficient evidence was found for long-term disability [30]. When comparing the public and private sectors, insufficient evidence was found for the association between the sector of employment and continued employment [22]. Furthermore, inconsistent evidence was found for the association between sector of employment and return to work [37, 47, 59, 63–68]. No studies were found for long-term disability with regard to sector.

## Discussion

In this systematic literature review, we explored the determinants at employer level associated with continued employment, return to work, and long-term work disability of workers with disabilities. Our findings indicate that organizational efforts on both supervisor level (i.e., work accommodations, support) and higher organizational levels (i.e., culture, policy), as well as company characteristics (i.e., sector, company size) can influence these work outcomes. At supervisor level, strong evidence was found for work accommodations. In addition, weak to moderate evidence was found for social support. Evidence for employer efforts at higher organizational levels was weak. Evidence for an association between company characteristics and continued employment, return to work and long-term disability was inconsistent.

### Supervisor Level: Work Accommodations

At supervisor level, our findings indicate that providing work accommodations is positively associated with continued employment and return to work, and negatively with long-term disability. The strength of evidence differed between work accommodation categories and the three work outcomes. We found strong evidence for the benefits of work accommodations concerning adaptations to work schedules for return to work, such as having the option to choose for flexible working hours [34] and to reduce working hours [28, 33]. We also found strong evidence for work accommodations concerning workplace adaptations, like the provision of a laptop computer that allowed workers to work from home [28], and changes in furniture at the office or workstation [27, 28, 33]. Moreover, we found strong evidence for work accommodations concerning interventions that aim to provide workers with additional support and guidance associated with return to work [26, 28, 33, 35–38]. These interventions focused on providing a workplace-oriented rehabilitation program like vocational work training or educational training, but also on providing occupational health services and case management interviews. We found moderate evidence for work accommodations regarding employer-provided changes in work in relation to continued employment [21–23, 32] which consisted of modifications to either work activities and duties [21, 23, 32] or the offer of a new job in the same company [22]. Additionally, we found moderate evidence for an association between employer-provided disability insurances [20, 69] and continued employment. For long-term work disability, we found insufficient evidence for work accommodations, which can be explained by the low number of articles available for this outcome.

The finding that offering work accommodations facilitates work participation is in line with previous reviews that reported on the evidence for adaptations to work schedules, providing equipment and modifications to work activities [6, 10, 16, 70–73]. However, most reviews studied work accommodations in relation to returning to work after sickness absence, but did not consider associations with continued employment and long-term work disability. For example, we found evidence that modifications to work activities are not only helpful for workers returning to work [73], but are also important in the context of staying employed after the onset of work disability. Our findings are consistent across different causes of work disabilities.

### Supervisor Level: Social Support

We found moderate evidence that social support from supervisors was related to return to work. Social support was operationalized as supervisor support as perceived by the worker [49–52, 54], a positive relation between supervisor and worker [53] and the supervisors’ communication with and response to workers [40, 46]. We found weak evidence for an association of social support from supervisors with continued employment [32, 45], which may be explained by the low number of included studies on this outcome. There were no articles included with long-term work disability as outcome.

The finding that social support facilitates work participation is consistent with several reviews [74–76] which found moderate-to-strong evidence for a positive relation between supervisor support and a shorter duration of sick leave, and reduction of workplace disability. However, two previous reviews on return to work, found no evidence for a positive relation of social support with return to work (yes/no) [77, 78]. This may be explained by the lower number of studies included in those return to work reviews compared to our study, as a consequence of these studies focusing on a specific disease group (e.g. cardiovascular disease and mental health). Compared with these two prior reviews, our review adds evidence concerning particular relational aspects of social support that are relevant for work participation of workers with all kind of work disabilities.

### Organizational Level: Culture

At organizational level, we found weak evidence for a positive association between organizational culture and return to work. Organizational culture includes a variety of determinants regarding the nature of the organizational culture (e.g. a people oriented culture, process or result oriented culture, open or closed culture, reward system, justice within an organization) [57–59], as well as determinants regarding organizational policies and practices (e.g. disability management programs and ergonomic policies) [52, 56]. No articles were included with either continued employment or long-term work disability as outcome.

There are some reviews on policies and practices (e.g. workplace disability management programs) that found insufficient evidence for an association with return to work [79, 80]. These reviews concluded that conclusions could not be made due to lack of evidence and high risk of bias in their included studies. Overall, more research on this topic is needed, as only a few studies could be included in our review. Moreover, there is a large variety in measurement of organizational culture across studies, as culture seems difficult to capture in questionnaires [81].

### Comparison of Findings Between Types of Diseases

In this systematic review, we included studies on workers with a broad range of disease groups. Because we included studies with different diseases we could provide an overview of prognostic factors that are relevant across different diseases, without specifically studying for differences between the disease groups. In almost half of these studies, the study population was defined as workers with work-limiting health conditions, i.e. all kinds of disability types were included and no distinction was made between the types of diseases. These studies were often found in the economic database. In contrast, studies from the field of medicine, occupational health and psychology often focused on a specific disease group, and included workers with a specific disability type, like mental health [35, 40, 48, 53, 58, 65, 66, 68], musculoskeletal disorders [27, 33, 46, 56, 67], and cancer [20, 25, 29, 34, 44, 45, 62].

Comparison of the studies showed that studies including workers with work-limiting health conditions mainly focused on the employer-domains work accommodations and company characteristics. For the disease-specific studies, we found that studies on mental health mostly focused on social support and company characteristics, whereas studies on musculoskeletal disorders and cancer mainly focused on work accommodations and company characteristics.

Comparison of the evidence showed that all studies including workers with work-limiting health conditions found positive evidence for an association between social support and work [47, 50, 51], whereas seven out of eleven studies on specific disease groups, like mental health, musculoskeletal disorders and cancer, found insignificant evidence for this association [32, 40, 44–46, 48, 49, 52–55]. We did not find any differences in evidence for specific work accommodations between the disease groups, nor between the specific disease groups in relation to the outcomes. This is in line with a previous study on supervisor competencies for supporting return to work following absence due to a mental health condition or a musculoskeletal disorder that showed that supervisor competencies relevant for return to work did not differ between workers with different chronic diseases [82]. Due to the low number of included studies on organizational culture, it was not possible to further analyze these findings. For the domain company characteristics, most studies found insignificant or even inconsistent evidence. For this reason, differences between generic and disease-specific studies and between disease groups were not studied.

### Strengths and Limitations

A strength of this review is that we included determinants of work participation at both supervisor level and organizational level. This provides a comprehensive overview of relevant employer determinants on different employer levels, in which context both the supervisor and organizational level plays a role.

Another strength of this review is that we only included longitudinal quantitative studies, which allowed us to summarize the evidence of the associations between the employer determinants and the work outcomes. However, the decision to exclude studies with a qualitative design entails that we excluded studies that could have provided more in-depth information about determinants like organizational culture and policies and practices.

Moreover, a strength of this review is the interdisciplinary perspective. Every included scientific field had their own contribution to our research topic. The economic studies primarily focused on continued employment, while medical and occupational health studies focused more on the return to work outcome. In the economic literature, the scope of studies was mostly on work accommodations and company characteristics, whereas the medical field focused on all the different employer domains. Furthermore, the economic studies mostly included data related to workers with work-limiting disabilities, whereas the medical, psychological and occupational health studies generally used data related to workers of specific disease groups. The inclusion of studies from these different fields enabled us to compare different outcome measures. The large consistency of the findings across the different outcome measures, makes us more confident about the strength of the presented evidence in our review, but also illustrate the added value of our interdisciplinary approach.

This study also has some limitations. In the field of economics it is common to publish working papers of submitted manuscripts because of the relatively long publishing process. In consequence of the decision not to include working papers we might have missed relevant recent papers from the economic perspective. Furthermore, we excluded studies in languages other than English and all included studies were from high-income countries. Consequently, we might have missed some useful studies from non-western countries, which may restrict the generalizability of the findings.

### Implications for Practice and Future Research

This review supports the assumption that the employer has a role in work participation of workers with disabilities. In particular, various work accommodations and supervisor support were found to be important for return to work and continued employment. However, for some work accommodations, like change of employer, job change, and professional assistance at- and outside of work, more research is needed on the impact on continued employment, return to work and long-term disability. Additionally, although supervisor support is a consistent determinant across the studies, further quantitative research is needed on supervisor support, which may include other aspects of social support, like instrumental or emotional support. Future research should therefore focus on the association between work outcomes and aspects of social support that have been found to be important in other studies. In this study, we cannot draw strong conclusions on the influence of culture and policies and practices due to the limited number of studies on organizational culture and organizational policies and practices, and the inconsistent measurement of organizational culture. Similarly, we found inconsistent evidence for company characteristics, which might be due to different classifications of company size and sector of employment. As organizational culture, policies and practices, and company characteristics could be important facilitators for employer support, further research is needed on the influence of these higher organizational levels on continued employment, return to work and long-term disability. Especially, more research is needed on how to measure the aspects of organizational culture that may be relevant for continued employment, return to work and long-term disability.

## Conclusion

This systematic literature review including studies from the economic, medical, psychological and occupational health field shows that employer support enables workers with disabilities to continue employment and return to work or reduce the likelihood of long-term work disability. Employer support entails organizational efforts on supervisor level and organizational level, as well as the role of company characteristics. This review especially shows positive evidence for the facilitation of work accommodations and for support of supervisors in relation with the above mentioned work outcomes. The evidence seems to be valid across studies that focused on specific and generic disease groups. Despite the weak evidence for organizational culture and inconsistent evidence for company size and sector of employment, our review indicates the importance of employer efforts on different organizational levels for preventing early labor market exit of workers with poor health. We found consistent evidence for a positive effect of efforts on supervisor level on the work participation outcomes. The role of organizational culture is less clear due to a weak level of evidence. However, as organizational culture is found to be important in qualitative studies, more research is needed on factors related to this concept. In this context, it is important for future longitudinal studies to achieve more consensus on the measurement of social support and organizational culture and policies.

## Supplementary Information

Below is the link to the electronic supplementary material.

Supplementary file1 (PDF 95 KB)
