# Supplementary material for: The Role of the Employer in Supporting Work Participation of Workers with Disabilities: A Systematic Literature Review Using an Interdisciplinary Approach
Source: J Occup Rehabil. 2021 May 12;31(4):916–49. doi: 10.1007/s10926-021-09978-3 (PMC8558169; doi:10.1007/s10926-021-09978-3)
Supplement: Supplementary file 1 — Supplementary file1 (PDF 95 KB) [file 10926_2021_9978_MOESM1_ESM.pdf]

Suplementary file

Search strategy Pubmed

#1 ("Chronic Pain"[Mesh] OR "Cardiovascular Diseases"[Mesh] OR "Diabetes Mellitus"[Mesh] OR "Fatigue Syndrome, Chronic"[Mesh] OR "Muscular Diseases"[Mesh] OR "Joint Diseases"[Mesh] OR "Rheumatic Diseases"[Mesh] OR "Multiple Sclerosis"[Mesh] OR "Pulmonary Disease, Chronic Obstructive"[Mesh] OR "Back Pain"[Mesh] OR "Neoplasms"[Mesh] OR "Asthma"[Mesh] OR "Headache Disorders, Primary"[Mesh] OR "Digestive System Diseases"[Mesh] OR "Nervous System Diseases"[Mesh] OR "Anxiety Disorders"[Mesh] OR "Bipolar Disorder"[Mesh] OR "Cyclothymic Disorder"[Mesh] OR "Depressive Disorder, Major"[Mesh] OR chronic illness*[tiab] OR chronic disease*[tiab] OR chronic pain[tiab] OR cardiovascular disease* [tiab] OR diabetes[tiab] OR chronic fatigue syndrome[tiab] OR musculoskeletal disease*[tiab] OR rheumatic disease*[tiab] OR pulmonary disease*[tiab] OR back pain[tiab] OR back problem[tiab] OR cancer[tiab] OR COPD[tiab] OR asthma[tiab] OR heart diseas*[tiab] OR depression[tiab] OR anxiety disorder*[tiab] OR disorder*[tiab] OR disease*[tiab] OR disabled*[tiab] OR diagnosis[tiab] OR impairment[tiab])

#2 ("Organizational Culture"[Mesh] OR "Organizational Policy"[Mesh] OR "Workplace"[Mesh] OR workplace*[tiab] OR work environment[tiab] OR organizational culture[tiab] OR organizational policy[tiab] OR organizational support[tiab] OR organisational culture[tiab] OR organisational policy[tiab] OR organisational support[tiab] OR employer*[tiab] OR supervisor*[tiab] OR worksite*[tiab] OR work accommodation[tiab] OR Human resource manage*[tiab] OR case manageme*[tiab])

#3 ("Return to Work"[Mesh] OR "Rehabilitation, Vocational"[Mesh] OR "Absenteeism"[Mesh] OR "Sick Leave"[Mesh] OR "Presenteeism"[Mesh] OR return to work[tiab] OR back to work[tiab] OR continuing work[tiab] OR continued work*[tiab] OR vocational rehabilitation[tiab] OR work ability[tiab] OR work participation[tiab] OR "Work Engagement"[Mesh] OR job retention[tiab] OR early retirement[tiab] OR labor force exit[tiab] OR job exit[tiab] OR absenteeism[tiab] OR sick leave[tiab] OR work absence[tiab] OR work disability[tiab] OR employment outcome*[tiab] OR work productivity[tiab] OR labor participation[tiab] OR labour participation[tiab] OR labor supply[tiab] OR labour supply[tiab] OR wage[tiab])

Searchstrategy Web of Science

TS="chronic pain" OR TS="diabetes mellitus" OR TS="fatigue syndrome" OR TS="multiple sclerosis" OR TS="back pain" OR TS="cancer" OR TS="asthma" OR TS="depression" OR TS="chronic illness" OR TS=disorder* OR TS=disease* OR TS=disabled OR TS=diagnosis OR TS=impairment

AND

TS="organi?ational culture" OR TS="organizational policy" OR TS="organizational support" OR TS="work environment" OR TS=workplace OR TS=employer* OR TS=supervisor* OR TS="work accommodation" OR TS=worksite OR TS="human resource manager" OR TS="human resource managers" OR TS="human resource management" OR TS="case management" OR TS=case manager OR TS=case managers

AND

TS="return to work" OR TS="vocational rehabilitation" OR TS=absenteeism OR TS="sick leave" OR TS=presenteeism OR TS="back to work" OR TS="continuing work" OR TS="continued work" OR TS="work ability" OR TS="work participation" OR TS="work engagement" OR TS="job retention" OR TS="early retirement" OR TS="labor force exit" OR TS="job exit" OR TS="work absence" OR TS="work disability*" OR TS="employment outcome" OR TS=work productivity OR TS=labo?r participation OR TS=labo?r supply OR TS=wage

Search strategy PsycINFO

#1 DE: "Chronic Illness" OR "Chronic Fatigue Syndrome" OR "Chronic Mental Illness" OR "Chronic Pain" OR "Diabetes Mellitus" OR "Musculoskeletal Disorders" OR "Multiple Sclerosis" OR "Cardiovascular Disorders" OR "Lung Disorders" OR "Back Pain" OR "Rheumatoid Arthritis" OR "Neoplasms" OR "Asthma" OR "Chronic Obstructive Pulmonary Disease" OR "Mental Disorders" OR "Digestive System Disorders" OR "Nervous System Disorders" TI and AB: "chronic illness" OR "chronic pain" OR "diabetes mellitus" OR "chronic fatigue syndrome" OR "multiple sclerosis" OR "back pain" OR asthma OR "cancer" OR copd OR disease* OR disorder* OR disabled OR diagnosis OR impairment

#2 DE: "Organizational Climate" OR "Working Conditions" OR "Employer Attitudes" OR "Management Personnel" OR "Human Resource Management" OR "Case Management" TI and AB: "organi?ational culture" OR "organi?ational policy" OR "organizational support" OR "work environment" OR workplace

OR employer* OR supervisor* OR "work accommodation" OR "worksite" OR "Human Resource Manage*" OR "case manage*"

#3 De: "Reemployment" OR   "Vocational Rehabilitation" OR   "Employee Absenteeism" OR "Employee Leave Benefits" OR   "Retirement" TI and AB: OR “return to work” OR “vocational rehabilitation” OR absenteeism OR “sick leave” OR presenteeism OR “back to work” OR “continuing work” OR “work ability” OR “employment outcome*” OR “work participation” OR “work engagement” OR “job retention” OR “early retirement” OR “labor force exit” OR “job exit” OR “work absence” OR “work disability” OR “employment outcome” OR “work productivity” OR “labo?r participation” OR “labo?r supply” OR “wage”

Search strategy Econlit

#1 TI and AB: “chronic illness” OR “chronic pain” OR “diabetes mellitus” OR “chronic fatigue syndrome” OR “multiple sclerosis” OR “back pain” OR asthma OR “cancer” OR copd OR disease* OR disorder* OR disabled OR diagnosis OR impairment

#2 TI and AB: "organi?ational culture" OR "organi?ational policy" OR "organizational support" OR "work environment" OR workplace OR employer* OR supervisor* OR "work accommodation" OR "worksite" OR "Human Resource Manage*" OR "case manage*"

#3 TI and AB: OR “return to work” OR “vocational rehabilitation” OR absenteeism OR “sick leave” OR presenteeism OR “back to work” OR “continuing work” OR “work ability” OR “employment outcome*” OR “work participation” OR “work engagement” OR “job retention” OR “early retirement” OR “labor force exit” OR “job exit” OR “work absence” OR “work disability” OR employment outcome OR work productivity OR labo?r participation OR labo?r supply OR wage
